# On Life

**Published:** 1827-11

**Authors:** George Smith Gibbes


### PHYSIOLOGY.

### *On Life.* By Sir George Smith Gibbes.

It appears, from well-established experiments, that all the animal tissues are resolvable on decomposition into minute bodies, which, in water, and under the influence of the sun, possess life and activity. These animalcula, or, more properly speaking, these ultimate points of vital activity, cannot be further decomposed except by the agency of fire, when they become subject to chemical laws, and assume the state of gas.

By the aid of the microscope and with a little management, it may be clearly seen that many of the processes of life depend upon these minute animals, and that the ordinary laws of matter, or the laws which regulate the material world, are totally out of the question in explaining the phenomena presented by these, the apparent rudiments of vitality.

The vitality and activity of the animalcula infusoria depend upon the influence of the sun, under which every pool of water becomes tenanted by myriads of them, all displaying, when examined by the microscope, the most unequivocal proofs of life.

The sun, the source of life as well as light, supplies this vitality in all the endless variety of organised and living action; and modifies matter, in all these processes, in a manner totally different from all physical and chemical principles. We might as reasonably compare a scarlet colour with the sound of a trumpet, as the phenomena of life and organisation with any of the subjects or any of the laws of the material world.

The most subtle fluids, as heat, light, electricity, magnetism, &c. present phenomena which every new discovery brings nearer in affinity to the material world. Life, on the contrary, is wholly independent of all these, opposing the laws of matter in every instance, and defying, in all its combinations, those laws of affinity and attraction which form the foundation of the physical sciences. Organised bodies are endowed with properties totally different from all others, and no portion of such bodies is subject to the ordinary laws of nature until every vestige of life be extinct.

Although in the dissecting room the human subject be dead as regards the creature then under consideration, yet the vitality is not lost, for every part of the organised structure resolves itself into new arrangements, and myriads of vital rudiments reassert their rank in the living world. Thus manures supply them to the growing vegetable, and digestion prepares them for the use of animals. Built up as the human fabric is by innumerable myriads of living rudiments, we easily admit the fact that every portion of it possesses vital powers: powers, in every possible view of them, wholly differing from the laws which regulate every subject of the material world.

Above thirty years ago I instituted a series of experiments respecting this very curious subject, which appeared then, as they now do, quite conclusive as to the essential purpose which the animalcula infusoria perform in the growth of vegetables. It was from considering the opinions of Ingenhouz,

Priestley, and others, on the nature of the green matter which forms on water, that I was led to examine very carefully with the microscope the animalcula infusoria, and to observe this matter, and the fibrillæ of the roots of other vegetables, whilst growing in water. Myriads of animalcula may be seen around the extremities of such vegetables, and it appears that these minute living bodies agglutinate themselves together, and *absolutely themselves become the added part*: so that the fibres seem to be nothing more than a congeries of these animalcula, forming the growing part. They may be seen like bees entering a hive, and making up, when fixed together, the fibre itself.

The whole substance of the conferva rivularis certainly appears to be nothing more than a condensed congeries of the animalcula infusoria. If a bason of water be half shaded from the sun, whilst the other half is exposed to its rays, we find the shaded water to be without the animalcula, whilst they swarm by myriads in the exposed portion.

If a sprig of mint be placed in this water, the fibres of the roots extend and grow in the illuminated portion, but they make no advance in the dark part. The animalcula are seen to be supplied on the one side, and to fix themselves on the ends of the fibres, and to increase them longitudinally: on the other side, the animalcula being absent, the roots do not grow. The increase of the several parts of vegetables seems entirely dependent on the supply which they receive of these animalcula by the roots, leaves, &c.; for the leaves and blades of corn, even when growing in a room, are terminated by drops of water evidently supplying these monades, which arrange themselves according to the necessities of the growing vegetable, and according to the impulse originally given, and continually supplied, by the seed of the plant.

The whole history and nature of compost and manure lead to the conclusion that, by certain decompositions of animal and vegetable matters, these first rudiments of life are again set free to become, under new arrangements, subservient to the growth of the renewed vegetation.

I purpose in my next communication to show the application of the foregoing observations to the animal system; their importance in explaining and directing the actions and functions of its most essential organs, and proving how far we may consider the living world independent of the ordinary laws of matter.

*Bath; September*, 1827.

